# Validity of self-reports of knee-straining activities at work: a field study with 6-month follow-up

**DOI:** 10.1007/s00420-012-0758-4

**Published:** 2012-03-18

**Authors:** Dirk M. Ditchen, Rolf P. Ellegast, Bernd Hartmann, Monika A. Rieger

**Affiliations:** 1 Institute for Occupational Safety and Health of the German Social Accident Insurance, Sankt Augustin, Germany; 2 Occupational Health Service of the Statutory Accident Insurance for the Construction Sector, Hamburg, Germany; 3 Institute of Occupational and Social Medicine, University Hospital of Tuebingen, Tuebingen, Germany; 4 Department of Occupational Health and Environmental Medicine, Institute of General Practice and Family Medicine, University of Witten/Herdecke, Witten/Herdecke, Germany

**Keywords:** Exposure assessment, Method comparison, Kneeling and squatting, Field study, Knee pain

## Abstract

**Objectives:**

To measure short-term and long-term validity of self-reported duration of kneeling and squatting at work and to examine the possibility of differential misclassification due to knee complaints.

**Methods:**

Work-related kneeling and squatting were analysed for 190 male subjects (mean age, 35.0 and SD, 11.5) in field by both measurements and questionnaires. Posture capturing was performed with an ambulatory measuring system (CUELA). Immediately after the measurement (*t*_0_), each participant was asked to estimate frequency and duration of five specific knee postures taken during the measurement period. After 6 months (*t*_1_), the survey was repeated (*n* = 125). Health status of all subjects was recorded by Nordic questionnaire. Statistical analysis was performed by using nonparametric tests, correlations, and Bland–Altman plots.

**Results:**

At both time points, subjects were able to recall the occurrence of knee postures rather well (100.0–57.6 % agreement) but many of them failed in quantifying their knee load. We found poor-to-moderate correlations between measurements and self-reports for all examined postures in both surveys (0.23 < ρ < 0.63). The durations of knee postures were both over- and underestimated but overestimations predominated (*t*_0,_ 74.7 % and *t*_1,_ 87.2 % overestimations). High-exposed subjects seemed to misjudge their exposure to a greater extent than low-exposed ones, while knee complaints seemed to have no impact on the assessment behaviour.

**Conclusions:**

As our study showed, self-reported knee loading may deviate widely from measured exposure. These limitations of self-reporting emphasise the arguments in favour of using objective data whenever possible, for example by complementing self-reported occurrence of knee postures with quantitative measurement data.

**Electronic supplementary material:**

The online version of this article (doi:10.1007/s00420-012-0758-4) contains supplementary material, which is available to authorized users.

## Introduction

Work-related knee-straining activities such as kneeling or squatting are recognised as risk factors for knee pathologies such as knee osteoarthritis and meniscal tears, a correlation documented by numerous international studies, especially case–control studies (Coggon et al. 2000; Cooper et al. 1994; Jensen 2005; Klussmann et al. 2010; Manninen et al. 2002; Sandmark et al. 2000; Seidler et al. 2008). In these studies, the identification of cases or patients often is based on the elaborate medical examinations including radiography, and the exposure assessment is usually conducted by using self-administered questionnaires (Felson et al. 1991; Muraki et al. 2009; Vingard et al. 1991). This means that study participants have to estimate their daily amount of kneeling or squatting retrospectively, often for work shifts decades ago. Thus, the validity of the information gained by self-reporting is one major criterion for the quality of these studies. For several kinds of occupational exposures, there are a number of studies showing low validity of self-reporting and poor correlations with measuring or observation methods, for example manual material handling (Viikari-Juntura et al. 1996), postures of the upper extremities (Descatha et al. 2009; Hansson et al. 2001), and duration of computer use (Douwes et al. 2007; IJmker et al. 2008).

In contrast, in the field of work-related knee loading, comparatively few studies related to this topic can be found. Furthermore, their results are not consistent: Some studies showed good agreement between self-reported and observed amount of knee loading (Jensen et al. 2000; Pope et al. 1998), others found poor validity of self-reported quantified knee load (Baty et al. 1986; Bolm-Audorff et al. 2007; Burdorf and Laan 1991; Klußmann et al. 2010; Viikari-Juntura et al. 1996). As the focus of most of these studies was primarily not on the validity of self-reporting, there are some methodological limitations that must be taken into account: small sample size (Baty et al. 1986; Klußmann et al. 2010; Viikari-Juntura et al. 1996), comparison of short working sequences (Burdorf and Laan 1991; Jensen et al. 2000), or inadequate methods for objective exposure assessment with respect to dynamic knee-straining tasks, for example screening methods with observation intervals of 20 or 30 s, respectively (Burdorf and Laan 1991; Pope et al. 1998).

All these studies analysed workers’ self-reports given immediately after the examination, thus disregarding long-term effects as they appear in retrospective studies. Apart from such memory effects, certain personal circumstances may also have an influence on workers’ assessment behaviour (recall bias). For example, some studies seem to support the impact of musculoskeletal disorders related to the examined risk factors on patients’ ability to estimate their exposure exactly (Balogh et al. 2004; d’Errico et al. 2007). Patients may tend to overestimate their exposure in contrast to people without such disorders (differential misclassification bias).

For these reasons, the aim of the current study was to examine the validity of self-reporting of work-related knee loading (i.e. kneeling, squatting, and crawling) by comparing them to the results gained by objective measurement, by analysing a sufficient study sample with subjects from several occupations, by conducting a two-stage survey (survey with six-month follow-up), and by examining the possible influence of current knee complaints on the accuracy of assessment in order to find out whether they may lead to differential misclassification.

The study is based on a scientific report made on behalf of the German Social Accident Insurance to investigate occupational kneeling and squatting in different occupations (Ditchen et al. 2010).

## Methods

### Design and study sample

**Fig. 1:**
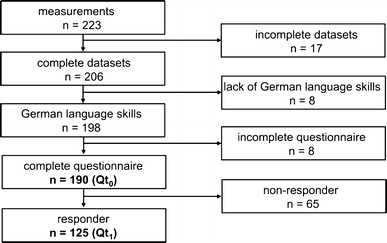
Recruitment of participants

As our study focussed on occupational knee loading in the construction and industrial sector, the following 20 occupations supposed to include knee-straining tasks were observed in this study (with numbers of subjects): installers (45), roofers (29), painters and decorators (20), tilers (19), parquet layers (19), screed layers (8), floor layers (9), pavers (7), reinforcing ironworkers (6), shipyard workers (5), mould makers (4), stone layers (4), tarp makers (4), welders (3), pipe layers (3), truck mechanics (2), electricians (1), steel builders (1), and assemblers (1). Recruitment of the 110 participating companies was conducted by members of the responsible social accident insurance. As study participants, 223 male craftsmen volunteered for field measurements. All of them were fit for work. For the current analysis, 33 data sets had to be excluded because of incomplete data sets (e.g. malfunction of video or measuring system), incomplete questionnaire, or lack of German language skills (Fig. 1), so 190 (=85.2 %) subjects remained for initial assessment. Their mean age was 35.0 years (SD, 11.5), and their mean duration of employment in the current occupation was 14.6 years (SD, 11.1). Information on health status was collected using a modified version of the Nordic questionnaire (Kuorinka et al. 1987). Six months later, 125 subjects participated in a second survey (Fig. 1).

### Posture capturing

Posture capturing was performed between October 2006 and June 2009 directly at the workplaces with the proprietary-developed measuring system CUELA (Ellegast and Kupfer 2000; Freitag et al. 2007; Glitsch et al. 2007). The mechanical-electronic system consists of gyroscopes, inclinometers, and potentiometers that can be fixed on a subject’s clothes with a belt system. The present version allows time continuous recording of body angles and the calculation of postures and movements of the trunk and lower limb. Thus, the occurrence, frequency, and duration of five different knee postures (unsupported kneeling, supported kneeling, sitting on heels, squatting, and crawling) for each subject were continuously measured and ready for analysis. A simultaneous video documentation completed the measuring setup. The average duration of a single measurement was about 2 h (mean, 118 min and SD, 44).

### Self-reports

#### Survey t_0_

Immediately after the measurement, each study participant was asked to fill out a short, printed questionnaire (*Qt*_0_) containing four questions about manual material handling, climbing stairs, jumping, and knee-straining postures occurring during the previous measurement. These postures were illustrated by five icons according to the legal definition of the German occupational disease No. 2112 “Knee osteoarthritis” (BMAS 2010). The question applied was previously used and pre-tested in a German study on workers’ assessment behaviour with regard to duration of knee-straining working activities (Klußmann et al. 2010; see Appendix A in Supplementary Material). Participants were asked to fill out a questionnaire after measurement but were not informed about its content. For this first survey, no compensation was paid. For quantification of the knee loading, the information about number and mean duration of the single actions was computed. Incomplete questionnaires were excluded from analysis.

#### Survey t_1_

All subjects agreed to participate in a future survey. Thus, 6 months after the first survey, another questionnaire (*Qt*_1_) was mailed to them. This questionnaire was identical to *Qt*_0_ but was accompanied with some short information about the working tasks during the measurement at *t*_0_ (e.g. *tiling the floor of a church for two hours* or *installing carpets on a hotel corridor for 1* *h*). Again, it was emphasised that exposure assessment should only be related to the period of measurement, indicated as start, end, and duration (in minutes). Participants were compensated (20€) after returning the completed questionnaire. However, from 190 participants, only 125 responded (65.8 %) and were valid for analysis (Fig. 1).

Overall, there were 65 non-responders: 54 subjects gave no response (even after two reminders), five subjects could no longer be contacted, and six subjects subsequently refused to participate. The characteristics of these non-responders and responders are shown in Appendix B in Supplementary Material.

### Data analysis

The results of the measurements and the two surveys were analysed by means of descriptive statistics (median, mean, and standard deviation). Additionally, a comparison between the results of the two methods (inter-rater reliability) was conducted on the basis of nonparametric statistics as the data sets cannot be assumed to be normally distributed (Kolmogorow–Smirnow test, not shown). The Wilcoxon signed-rank test (paired samples) and the Spearman’s rank correlation coefficient (ρ) were calculated to find differences or correlations between self-reports and measurements. The correlation coefficients were interpreted as follows: very poor (ρ ≤ 0.2), poor (0.2 < ρ≤ 0.5), moderate (0.5 < ρ≤ 0.7), good (0.7 < ρ ≤ 0.9), and very good (ρ > 0.9) (Bühl and Zöfel 2000).

We calculated percentage of agreement in order to compare the different methods with respect to the pure identification of knee postures. In addition, we generated Bland–Altman plots (Bland and Altman 1986) using MedCalc (v 11.4.1.0, MedCalc Software bvba) to examine the proportion of over- and underestimations and the impact of different exposure levels on the accuracy of subjects’ self-reports. In order to detect a possible differential misclassification caused by knee disorders, we split the total sample into two subgroups (subjects with knee complaints in the last 12 months and subjects without such complaints) and applied the Mann–Whitney *U* test (for two independent samples). All statistical analyses were done using SPSS (v 18, SPSS Inc.).

## Results

### Identification of knee-straining postures

In both surveys, subjects were able to recall very well whether they performed knee-straining postures or not. At *t*_0_ (*n* = 190), there was total agreement between survey and measurement regarding the occurrence (no/yes) of any of the five knee postures (100 %) (Table 1, identification of knee loading). With respect to the several forms of knee postures, the percentage of agreement ranged between 67.4 % (squatting) and 90.0 % (unsupported kneeling).

**Table 1:** Identification and quantification of knee-straining postures within measurement (M) and both questionnaires (*Qt*_0_ and *Qt*_1_)

Postures	Identification of knee postures (percentage of agreement)		Duration of knee-straining activities (min)							
			Survey *t*_0_ (*n* = 190)				Survey *t*_1_ (*n* = 125)			
	*M* − *Qt*_0_	M − *Qt*_1_	*M*		*Qt*_0_		*M*		*Qt*_1_	
	(*n* = 190)	(*n* = 125)	Median (range)	Mean (SD)	Median (range)	Mean (SD)	Median (range)	Mean (SD)	Median (range)	Mean (SD)
Unsupported kneeling	90.0	87.2	15.3 (0.0–125.0)	20.9 (20.3)	20.0 (0.0–1,064.0)	52.8 (116.6)	17.2 (0.0–125.0)	22.8 (21.7)	20.0 (0.0–1,400.0)	76.4 (194.2)
Supported kneeling	85.8	81.6	2.9 (0.0–73.0)	9.2 (14.3)	11.0 (0.0–1,200.0)	44.9 (115.1)	2.6 (0.0–73.0)	10.5 (15.9)	25.0 (0.0–18,000.0)	316.3 (1,795.2)
Sitting on heels	71.6	76.8	1.4 (0.0–57.9)	4.2 (6.8)	1.5 (0.0–360.0)	16.7 (46.0)	1.8 (0.0–57.9)	4.5 (7.6)	11.0 (0.0–18,000.0)	193.8 (1,607.5)
Squatting	67.4	67.2	0.9 (0.0–83.4)	5.0 (11.5)	2.5 (0.0–300.0)	17.3 (37.8)	0.8 (0.0–78.6)	4.5 (10.2)	6.0 (0.0–2,000)	54.4 (204.5)
Crawling	73.2	57.6	0.0 (0.0–7.0)	0.2 (0.9)	0.0 (0.0–900.0)	19.2 (90.5)	0.0 (0.0–7.0)	0.3 (1.0)	2.0 (0.0–9,000.0)	121.7 (822.9)
Knee postures in total	100.0	95.2	32.7 (0.0–146.8)	39.3 (32.3)	60.0 (0.0–2,200.0)	152.2 (279.4)	33.9 (0.0–146.8)	42.6 (34.5)	105.0 (0.0–39,850)	762.6 (3,977.0)

Survey *t*_1_ (*n* = 125) resulted in a high percentage (95.2 %) of agreement between subjects’ assessment and measurement for the occurrence of any knee posture, as well, showing a range from 57.6 % (crawling) to 87.2 % (unsupported kneeling) for the single knee postures.

### Quantification of knee loading

The proportion of knee-straining postures during the measuring period over all 190 measurements was 34.1 % (SD, 24.7 %) and the coefficient of variability (CV) was calculated to 0.72.

The quantitative assessment of knee loading obtained by self-reports and measurement is presented in Table 1 (duration of knee loading). In contrast to the good agreement found in identifying knee postures, comparing the quantification of knee load assessed by both methods showed considerable differences between questionnaires and measurement.

In survey *t*_0_, the median duration of the reported *knee postures in total* was about twice as high as the corresponding measured result (60.0 compared to 32.7 min). Regarding the median duration of the single kinds of knee postures, the duration of knee postures seemed to be overestimated by the participants (e.g. *supported kneeling* 11.0 compared to 2.9 min, *squatting* 2.5–0.9 min), while the agreement between the median results of measurements and self-reports for *sitting on heels* and *crawling* was good (1.4 compared to 1.5 min and 0.0–0.0 min, respectively). Obviously, the self-reported durations of knee postures varied to a far greater extent than the corresponding measured results (e.g. standard deviation *knee postures in total* 279.4 compared to 32.3 min). Moreover, extreme and implausible overestimations for all examined postures occurred to a high degree: Self-reported mean durations of knee postures exceeded the mean measurement results many times over (e.g. *knee postures in total*, 152.2 compared to 39.3 min, *supported kneeling*, 44.9–9.2 min).

These findings could be confirmed for survey *t*_1_, where, for example, the median self-reported duration of *knee postures in total* was about three times as high as the corresponding measured duration (105.0 compared to 33.9 min), while the differences between the self-reported and measured median durations of the single knee postures ranged from nearly no difference (*unsupported* *kneeling,* 20.0 compared to 17.2 min) to slight (*crawling,* 2.0–0.0 min) to serious overestimation (*supported* *kneeling,* 25.0–2.6 min). Again, the reported durations showed huge variations compared with those of the measured results for all examined postures (e.g. standard deviation *knee postures in total*, 3,977.0 compared to 34.5 min SD) and extreme values with a high impact on the arithmetic mean values (e.g. 762.6 compared to 42.6 min for the *knee postures in total*).

### Rank sum test and correlation

The results of the nonparametric statistics are presented in Table 2. The already observed differences between self-reports and measurements are affirmed by the results of the Wilcoxon signed-rank test (paired samples), which shows highly significant differences between both methods in all examined postures—both for survey *t*_0_ and survey *t*_1_.

**Table 2:** Results of the Wilcoxon signed-rank test (paired samples) and the Spearman’s rank correlation coefficient for the duration of knee-straining activities comparing measurement and the results of the surveys *Qt*_0_ and *Qt*_1_ (numbers in parentheses represent *p* values for the Spearman’s correlation coefficients)

Postures	Measurement compared to survey *t*_0_ (*n* = 190)			Measurement compared to survey *t*_1_ (*n* = 125)		
	Wilcoxon	Spearman’s correlation		Wilcoxon	Spearman’s correlation	
	*p*	ρ	95 % CI	*p*	ρ	95 % CI
Unsupported kneeling	0.0001	0.55 (<0.0001)	(0.45–0.65)	0.0160	0.28 (0.0007)	(0.11–0.44)
Supported kneeling	<0.0001	0.63 (<0.0001)	(0.54–0.71)	<0.0001	0.54 (<0.0001)	(0.41–0.66)
Sitting on heels	<0.0001	0.42 (<0.0001)	(0.29–0.53)	<0.0001	0.32 (0.0002)	(0.15–0.47)
Squatting	<0.0001	0.40 (<0.0001)	(0.27–0.51)	<0.0001	0.33 (<0.0001)	(0.16–0.48)
Crawling	<0.0001	0.42 (<0.0001)	(0.30–0.53)	<0.0001	0.23 (0.0013)	(0.06–0.39)
Knee postures in total	<0.0001	0.63 (<0.0001)	(0.54–0.71)	<0.0001	0.43 (<0.0001)	(0.28–0.57)

For Spearman’s rank correlation coefficient, we found poor-to-moderate correlations with the measurement data in both surveys: In survey *t*_0_, we calculated values between 0.40 (*squatting*) and 0.63 (*supported kneeling*), in survey *t*_1_, correlations ranged from 0.23 (*crawling*) to 0.54 (*supported kneeling*).

### Assessment behaviour and exposure level

With respect to absolute time of *knee postures in total*, survey *t*_0_ resulted in 142 overestimations (percentage of agreement, 74.7 %), 38 underestimations (20.0 %), and 10 agreements (5.3 %). The corresponding figures in survey *t*_1_ are 109 overestimations (87.2 %), 13 underestimations (10.4 %), and three agreements (2.4 %). Thus, overestimations (including implausible answers with regard to the duration of exposure as compared to the measurement period) predominate in survey *t*_0_ and even more strongly in survey *t*_1_, but in both surveys, underestimations were not negligible.

**Fig. 2:**
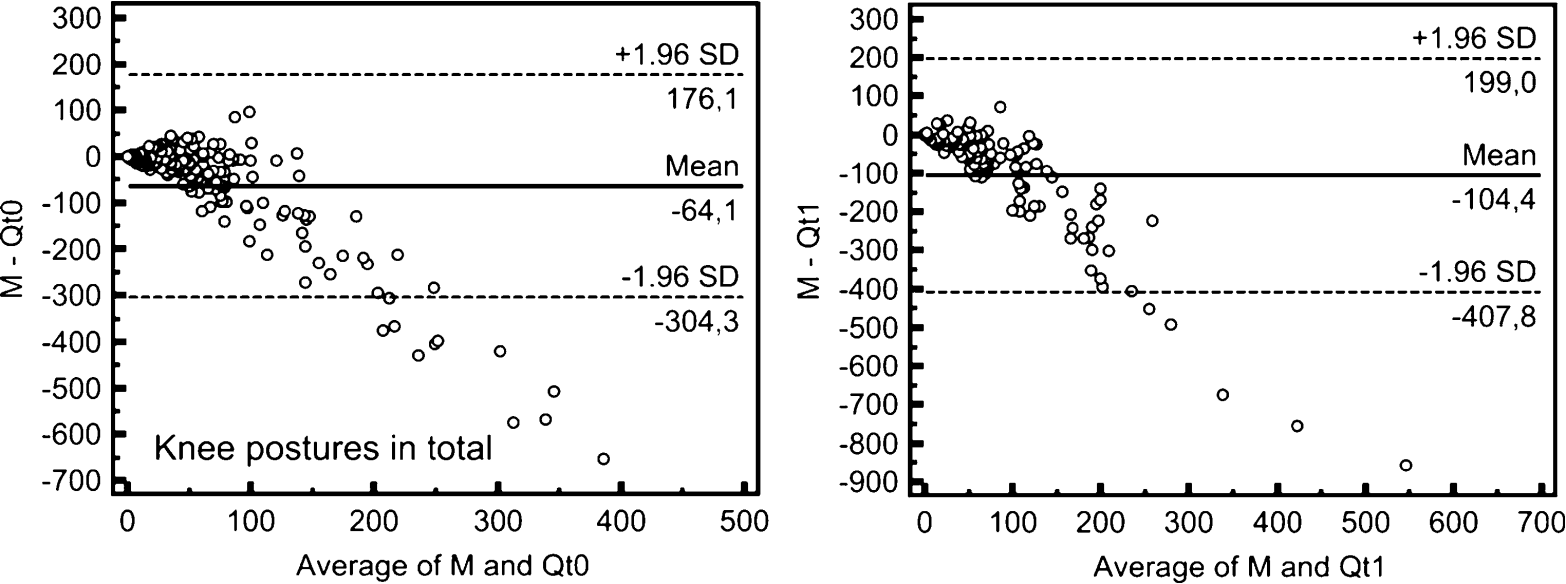
Bland–Altman plots for the comparison of both measurement and *Qt*_0_ (*left*) and *Qt*_1_ (*right*), showing knee postures in total [min]; *n*(t_0_) = 182, *n*(t_1_) = 116 (for better illustration, eight outliers (*Qt*_0_ > 1,000 min) and nine outliers (*Qt*_1_ > 1,000 min), respectively, were excluded)

This assessment behaviour can also be recognised in the corresponding Bland–Altman plots for both surveys (Fig. 2; positive values on the y-axis illustrate underestimations, and negative values describe overestimations; for better illustration, outliers as defined in the legend were excluded). Moreover, the plots show relatively good agreement between both methods within the range of missing or low exposure. With increasing exposure, however, agreement worsened. This effect is shown in the fan-shaped distribution of the data points relative to the coordinate origin. Obviously, the overestimations prevailed. This is documented by the negative values of mean in survey *t*_0_ (−112.9 or −64.1 min after excluding eight outliers, respectively) and survey *t*_1_ (−720.1 or −104.4 min after excluding nine outliers, respectively). In both surveys, the *limits of agreement* including about 95 % of the data (±1.96 SD) embrace a huge range of data. In survey *t*_0_, these limits range from −646.5 to 420.5 min (or from −304.3 to 176.1 min after excluding eight outliers, respectively), in survey *t*_1_, from −8,535.9 to 7,095.8 min (or from −407.8 to 199.0 min after excluding nine outliers, respectively).

**Fig. 3:**
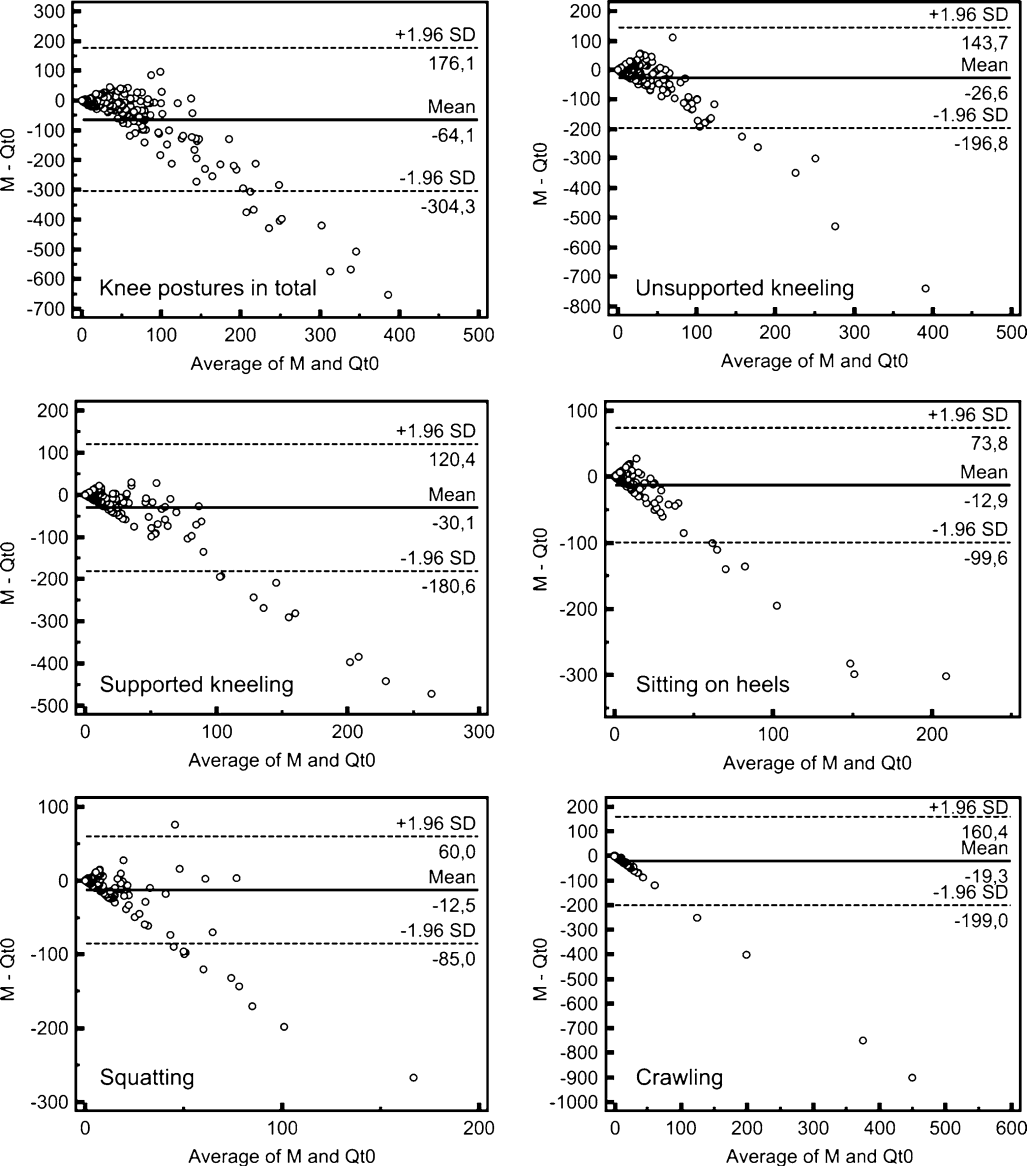
Bland–Altman plots for the comparison of measurement and *Qt*_0_, showing all examined knee postures [min] (for better illustration, outliers (*Qt*_0_ > 1,000 min) were excluded); sample sizes: knee postures in total (182), unsupported kneeling (189), supported kneeling (189), sitting on heels (190), squatting (190), and crawling (190)

Figure 3 shows Bland–Altman plots for all examined knee postures for the comparison of measurement and questionnaire *Qt*_0_. Except in the case of *crawling*, the results for all postures can be interpreted in a similar way as the *knee postures in total*: The means have negative values in all cases, and the limits of agreement show deviations of at least 60 min in both directions (over- and underestimation). On a low exposure level, good agreement between both methods can be stated but with increasing exposure, the deviations increased, as well. Overestimation predominated for all postures, but underestimation also occurred for all postures except *crawling*, which was always overestimated.

### Subjects with knee disorders versus subjects without knee disorders

A total of 182 of 190 participants in survey *t*_0_ filled out the Nordic questionnaire. Of these, 55 subjects (=30.2 %) reported knee complaints in the last 12 months (group k1), while 127 participants (=68.8 %) reported none (group n1). The comparison of assessment behaviour in the two groups was based on the differences between self-reported and measured durations of *knee postures in total* in both surveys. The Mann–Whitney *U* test for two independent samples showed no significant differences between the two groups (medians in groups k1 and n1 were 31.3 and 14.6 min, Mann–Whitney *U* = 3,026.5, *p* = 0.153 two tailed). Repeated tests in several subgroups of age, years in trade, and level of exposure showed no difference in the assessment behaviour of both groups.

In survey *t*_1_, details on health status for 119 of 125 subjects were available. Of these subjects, 38 (=31.9 %) reported knee complaints in the last 12 months (group k2) and 81 subjects (=68.1 %) comprised the “no complaints”-group (n2). The result of the Mann–Whitney *U* test was similar to survey *t*_0_ showing no significant differences (medians in groups k2 and n2 were −69.0 and −49.5 min, Mann–Whitney *U* = 1,355.0, *p* = 0.294 two tailed). Again, age, years in trade, and level of exposure seemed to have no impact on the assessment behaviour in both groups.

With respect to any musculoskeletal complaints in the last 12 months, we found similar results in both surveys (*t*_0_, *p* = 0.750; *t*_1_, *p* = 0.835).

## Discussion

### Validity of self-reports on knee loading

The present study showed two different aspects of self-reported knee load: good to acceptable quality in identifying knee postures but mostly poor to very poor quality in quantifying the load. These conclusions are supported by related studies on several musculoskeletal risk factors (Descatha et al. 2009; Stock et al. 2005; Unge et al. 2005) and knee loading in particular (characteristics of the referred studies are shown in Appendix C in Supplementary Material): In a Finnish study on forest industry workers, Viikari-Juntura et al. (1996) described a poor correlation between observed and self-reported amount of kneeling and squatting (Spearman’s ρ = 0.42, *p* < 0.001). Hence, they determined self-reports to be helpful in identifying high exposure groups but to be inappropriate in quantifying the exposure. Their results were based on the direct workplace observations of 36 workers, compared with self-reports on the exposure of an average work shift from 2,756 workers.

Baty et al. (1986) examined working postures of 46 nurses by observation and registration of major body postures every 15 s. At the end of the work shift, participants were asked to assess the amount of time spent in several postures. For kneeling and squatting, a good agreement between observed and self-reported occurrence was found (22/23 and 10/11 agreements, respectively), while the nurses overestimated their duration of kneeling and squatting four times on average. It should be kept in mind that kneeling and squatting postures occurred only infrequently.

In a Dutch study, 35 mechanical repairmen were observed at the workplace and asked to keep a log every hour to assess exposure to several musculoskeletal risk factors (e.g. kneeling/squatting) for a whole work shift (Burdorf and Laan 1991). Subjects were able to assess the occurrence of kneeling/squatting activities quite well, but the percentage of daily work time in these postures was slightly underreported.

In a German study, task analyses on 25 workers were carried out using an observational method (Klußmann et al. 2010). At the end of the work shift, 92 % of the subjects were able to report the occurrence of knee postures correctly but failed in quantifying their kneeling exposure (average deviation between self-reported and observed duration of kneeling, 171 %).

In another German study, 75 construction workers were observed for 4 h at the workplace, and their exposure to kneeling and squatting was quantified with a stop watch (Bolm-Audorff et al. 2007). After the observation, subjects were asked to assess the duration of kneeling and squatting postures during the observation. The results of the self-reports and the observation showed a good Pearson’s correlation (*r*² = 0.74, *p* < 0.01), but workers seemed to overestimate their knee load systematically: the median self-reported duration of knee postures was reported as 35 % of the working shift, while the median for the observations was 21.9 % (*p* < 0.001).

However, there are a few studies on this topic with contradictory results. In a British study with 123 participants from various occupations, the self-reported durations of kneeling postures taken directly after the examination agreed well with the observed amount of kneeling (Pope et al. 1998). This may be caused by the relative rare occurrence of kneeling activities (only about 50 % of the observed tasks included this exposure) and the observation method (recording of postures all 30 s during 1 h of working time), which may not be suited for quantitative measures of highly dynamic tasks. A Danish study on occupational knee loading in 33 floor layers and 38 carpenters also reported good correlations (Spearman’s ρ = 0.89) between self-reported and video-recorded amount of kneeling and squatting (Jensen et al. 2000). However, the examined working sequences were rather short (three to 30 min) and included very homogenous tasks, which may support a good recall of the knee load.

The variability of the studied exposure to knee-straining postures may also have an impact on the validity of assessment. In comparison with the referred studies above, our study sample must be seen as rather homogeneous in respect to knee-straining postures (CV = 0.72, cf. Appendix C in Supplementary Material) as we involved tasks in our study which were supposed to be knee-straining.

All reported studies examined only self-reports taken immediately after the exposure event or at the end of the working shift. In contrast, the present study was interested in subjects’ ability to assess their exposure a half-year later, as well. In this second survey, subjects’ ability to recall the occurrence of knee postures can be rated as acceptable to good. However, the validity of the self-reported durations of these postures was worse than in the first survey. To the best of our knowledge, there are no similar published studies on this topic.

### Assessment behaviour and impact of exposure level

In both surveys, participants tended to overestimate their exposure, especially in survey *t*_1_ (87.2 % overestimations). Nevertheless, underestimations can be observed in both surveys. Both phenomena have been reported in several studies on assessment of knee loading: clear overestimation (Bolm-Audorff et al. 2007), predominating underestimation (Burdorf and Laan 1991), and deviations in both directions in one sample (Jensen et al. 2000). Thus, the assessment behaviour may depend on the wording of the questionnaire, the study sample, or the exposure level (Barrero et al. 2009).

As this study indicates, exposure level seems to have an enormous impact on the validity of self-reported knee exposure. In both surveys, differences between reported and recorded durations of knee postures were small at a low exposure level but increased with increasing exposure. Participants were able both to report the absence of knee postures exactly and to assess short time exposure, especially by comparing absolute values (see Bland–Altman plots) rather than relative ones. On the other hand, high-exposed subjects were misjudging their amount of knee loading by far. Confirming this effect, a study on the duration of computer use of 87 computer workers reports comparable assessment behaviour for low- and high-exposed subjects (Heinrich et al. 2004). But in contrast, another study on that topic gives an opposite result: agreement between self-reported and observed duration of computer use of 572 office workers improved with increasing exposure (IJmker et al. 2008). This effect might be explained by the use of categorical data (seven response categories for hours of computer use per day), while we used continuous data for assessment in our study. With respect to occupational knee load, only one of the cited studies took assessment behaviour of low- and high-exposed subjects into consideration (Klußmann et al. 2010). In a sub-analysis of this study, high-exposed workers showed a better ability to assess their exposure than low-exposed. However, study sample was rather small (*n* = 25) and deviations between both methods were only reported as relative differences instead of absolute numbers; thus, the effect may be overestimated.

### Impact of knee disorders on the validity of self-reports

The present study gave no hint of a differential misclassification of exposure due to self-reported knee complaints. Participants both with and without such complaints showed comparable assessment behaviour. This result seems to be contrary to studies reporting differential misclassifications caused by several forms of musculoskeletal complaints and risk factors such as low back pain and manual material handling (Wiktorin et al. 1993), neck-shoulder complaints and awkward postures of head, back and arms (Hansson et al. 2001), or upper limb complaints, and physical activity (Balogh et al. 2004).

In terms of occupational kneeling or squatting, only a few studies considered the impact of musculoskeletal disorders on the assessment behaviour. Moreover, if complaints were taken into account, it was not about knee complaints. Burdorf and Laan (1991) found no impact of low back pain or shoulder pain on self-reported kneeling or squatting of mechanical repairmen. Sample size of that study was certainly small (*n* = 35) and kneeling or squatting just made an average of only 14 % (SD, 12) of the observed time in the sample. In contrast to that, Viikari-Juntura et al. (1996) reported an increased risk of reporting high workload for forest industry workers having severe low back pain, e.g. for kneeling and squatting (OR, 1.6; 95 % CI, 1.2–1.9). Again, sample size was small (18 subjects with and 18 subjects without low back pain), and squatting or kneeling was rare in both groups (median, 0.0 h each). As the present study has dealt with knee complaints, our results cannot be closely compared to those studies. Moreover, our study concentrated on kneeling or squatting tasks (median, 32.7 min or 29.7 % (0.0–92.7) of knee postures per measurement). With certain constraints, it should be noted that subjects with severe knee pain probably did not participate in our study due to sick leave.

### Study limitations

The present study has several limitations that should be considered when interpreting the results.

The study was based on the voluntariness of participation of companies and subjects, which might have led to selection bias. Moreover, we examined only tasks where we expected knee-straining postures. Thus, our results are not representative for the whole working content of the examined trades.

While in survey *t*_0_ all measured subjects filled out the questionnaire, in survey *t*_1,_ only 65.8 % of the participants responded. However, compared to response-rates of other studies in Germany, this can be seen as quite successful (Latza et al. 2004). A non-responder analysis yielded similar to identical characteristics for responders and non-responders (see Appendix B in Supplementary Material). This lack of difference suggests that the lost to follow-up may not be an important issue, and the risk of a non-responder bias may be ruled out.

As the second survey was conducted by mail, study participants were only able to ask comprehension questions in the first survey when study staff was on site. Thus, comprehension problems may have occurred in the second survey more often and may have biased the exposure assessment, for example by self-reported exposure wrongly related to a whole work shift, rather than to the measuring period. However, we attempted to minimise this effect by using the same questionnaire as in the first survey, accompanied by information on how to correctly fill it out. In addition, we gave a short description of the work performed during the exposure measurement at *t*_0_. This procedure could have artificially reduced recall bias as such information cannot be provided in an epidemiological study, for example.

Our survey covered a pre- and post-period of 6 months, while in reality, there are mostly several years or decades between exposure and retrospective assessment. Thus, the results of our study might not be transferred directly on the validity of long-term exposure assessment but may give a hint on how the validity of assessment will decrease in time.

The form of questions presented on the duration of knee postures may be critical, as participants had to quote frequency and duration of their postures and were not able to see the result of their total time in knee postures (unless they calculated it for themselves). For that reason, self-reported durations of knee postures even higher than the whole measuring period can be found in both surveys (33.7 % of all data in survey *t*_0_, 44.5 % in survey *t*_1_). This effect is also known for other studies using open-ended questions for exposure assessment (e.g. Douwes et al. 2007). As we were only interested in subjects’ assessment behaviour rather than in getting plausible self-reported information, we refrained from excluding implausible data from the analysis as is necessary in an epidemiological study. In order to recognise a possible bias caused by this, we performed a statistical sub-analysis including only data sets from survey *t*_0_ reporting total duration of knee postures within duration of measuring period. This sub-analysis showed no significant differences relative to the total sample. Furthermore, there were no significant differences in age, profession, education, or number of years in profession between subjects who reported extremely implausible duration of knee postures and subjects giving plausible self-reports.

Taking absolute time units as assessment units (*minutes*) may have caused problems, especially for short-term activities. But asking relative percentages of time seemed to be unsuitable as the measuring periods were not of constant duration but had to be applied to particular working situations. Furthermore, there are some hints that subjects may assess the duration of occupational tasks better in terms of absolute time than as percentage of time (Heinrich et al. 2004).

### Strengths

The main strength of this study is its examination of self-reports at two different time points to demonstrate the effect of recall bias on the validity of assessment. Most studies on method comparison have only been concerned with short-term validity of self-reports, as done in survey *t*_0_ of this study. Furthermore, we applied a highly valid and suitable measuring technique as criterion method. In a recent review on method comparison, this kind of reference method is described as being of the highest quality level (Barriera-Viruet et al. 2006).

Both questionnaire and measurement were compared “one to one”, that is, in both surveys, the two methods referred to identical subjects and time periods. Thus, time periods for the self-reports were well defined and matched to the measurement periods, which is also described as a criterion of high quality (Stock et al. 2005; Barrero et al. 2009).

Study samples in survey *t*_0_ (190 participants) and survey *t*_1_ (125 participants) must be regarded as large in comparison with related studies. In another recent review, mean sample size of the described ten studies was 104 (SD, 63) or 79 (SD, 30), respectively, for four studies also using measuring techniques as criterion method (Stock et al. 2005).

The additional registration of subjects’ health status allowed the examination of a possible differential misclassification due to knee complaints in assessing work-related knee loading, a relation—as we have found—not yet reported in the literature.

## Conclusions

As our study indicated, self-reports on work-related kneeling and squatting showed high validity in identifying the occurrence of these postures but mostly low validity in quantifying them. Thus, the results support the request for adequate measures of exposure assessment in epidemiological studies. The use of questionnaires undeniably offers a number of advantages such as low cost, wide-spread application, a great variety of different kinds of assessable exposures, and the survey of retrospective exposures. Nevertheless, their results must be analysed with care, as recall bias, or differential misclassification bias may have an enormous influence on the validity of these results. In this spirit, the study emphasises the question “In musculoskeletal epidemiology are we asking the unanswerable in questionnaires on physical load?” (Burdorf and van der Beek 1999). To avoid such problems, questionnaires in the field of work-related knee loading should be adequately applied, for example, to identify workloads or load concentrations, to evaluate preventive measures, or to assess perceived exertion. To quantify loading, it seems to be useful to combine questionnaires on tasks or the occurrence of knee loads with more valid quantitative data, for example measuring data, whenever possible. Similar approaches can be found in the field of chemical exposures (Semple et al. 2004). Furthermore, our study showed the importance of thorough correction for implausible self-reported information in epidemiological studies.

## Electronic supplementary material

Below is the link to the electronic supplementary material.

Supplementary material 1 (DOC 196 kb)
