# Supplementary material for: Validity of self-reports of knee-straining activities at work: a field study with 6-month follow-up
Source: Int Arch Occup Environ Health. 2012 Mar 18;86(2):233–43. doi: 10.1007/s00420-012-0758-4 (PMC3555247; doi:10.1007/s00420-012-0758-4)
Supplement: Supplementary file 1 — Supplementary material 1 (DOC 196 kb) [file 420_2012_758_MOESM1_ESM.doc]

# Appendix A: Questionnaire Qt_0_

| Subject No: | _ _ _ _ _ _ _ / _ _ / _ _ - _ _ - _ _ _ _ / P _ | | | | | |
| --- | --- | --- | --- | --- | --- | --- |
| Task(s): |  | Measuring period: | from |  | to |  |

**The assessment refers only to the measuring period.**

**Question 4:**

During today’s measurement: Have there been any activities with one or more of the following postures: **Kneeling**, **squatting,** **sitting** **on** **heels,** or **crawling**?

no  yes

If yes, please fill out this table.

| Posture | | Occurring? | Number of events | Average duration of one event (in minutes) |
| --- | --- | --- | --- | --- |
| 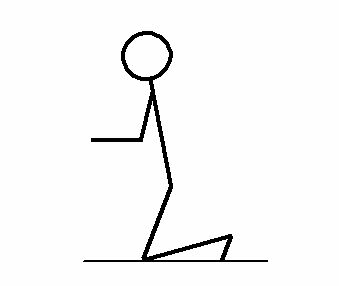 | Unsupported kneeling | no  yes | ..........times | ............min |
| 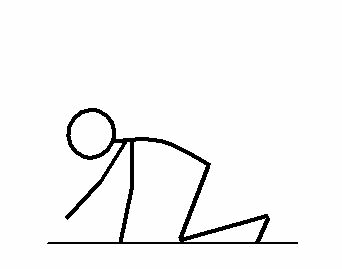 | Supported kneeling | no  yes | ..........times | ............min |
| 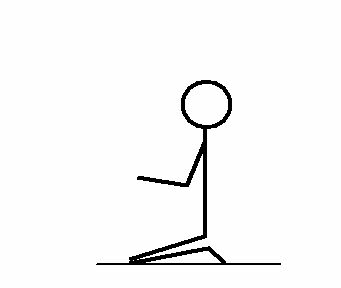 | Sitting on heels | no  yes | ..........times | ............min |
| 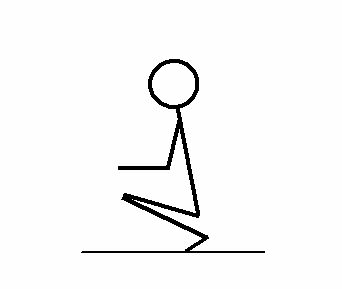 | Squatting | no  yes | ..........times | ............min |
| 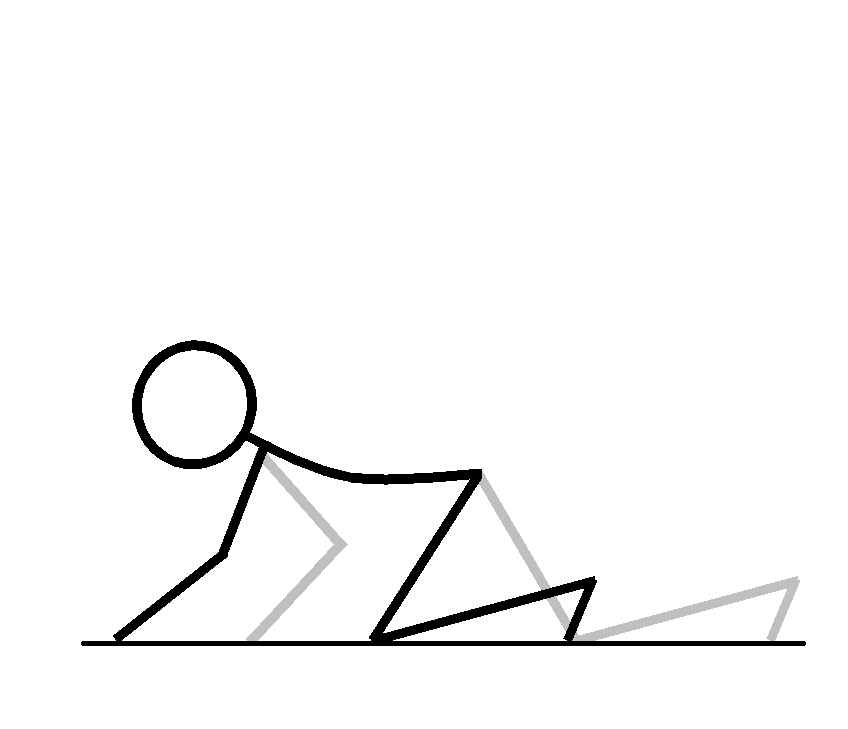 | Crawling | no  yes | ..........times | ............min |

# Appendix B: Characteristics of responders and non-responders t_1_

All study participants were male active craftsmen (blue-collar-workers) with sufficient German language skills. Values in table for age, years in trade and percentage of knee-straining postures are arithmetic means and standard deviations (in brackets).

|  |  | N |  | Age |  | Years in trade |  | Percentage of knee-straining postures (measurement) |
| --- | --- | --- | --- | --- | --- | --- | --- | --- |
| Responders t_1_ |  | 125 |  | 36.1 (11.9) |  | 15.9 (11.6) |  | 34.5 (23.5) |
| Non-responders t_1_ |  | 65 |  | 32.6 (10.3) |  | 12.0 (9.6) |  | 33.3 (27.0) |
| Participants t_0_ |  | 190 |  | 34.9 (11.5) |  | 14.6 (11.1) |  | 34.1 (24.7) |

# Appendix C: Characteristics of studies cited in “Discussion”

| Study |  | Included trades |  | Participants  (n, sex) Age (years) |  | Objective method(s) |  | Subjective method(s) |  | Kneeling/squatting  (Coefficient of variability) |
| --- | --- | --- | --- | --- | --- | --- | --- | --- | --- | --- |
|  |  |  |  |  |  |  |  |  |  |  |
| Baty et al. (1986) |  | Nurses |  | 46 females  n/a |  | Direct observation of major body postures (every 15 s), full work shifts |  | Interview at the end of the observed shift (incl. kneeling/ squatting: real time assessment, h and min) |  | 2.15 |
|  |  |  |  |  |  |  |  |  |  |  |
| Bolm-Audorff et al. (2007) |  | Construction workers (e.g. tilers, pavers, roofers, screed layers) |  | 75 males  n/a |  | Direct observation major body postures, continuously with stop watch over 4 h |  | Self-assessment at the end of the observation (incl. kneeling, squatting and sitting on heels as percentage of shift) |  | 0.90 |
|  |  |  |  |  |  |  |  |  |  |  |
| Burdorf and Laan (1991) |  | Mechanical repairmen (e.g. pipe fitters, benchmen) |  | 35 males  Mean 44 (SD 10) |  | OWAS-observation method (every 20 s) in two periods of 10 min covering all important work tasks |  | 1. Questionnaire for duration and frequency of basic work postures (incl. kneeling/squatting as h/day)  2. One day log with the same questions, filled out every hour on the observed shift |  | 0.86 |
|  |  |  |  |  |  |  |  |  |  |  |
| Jensen et al. (2000) |  | Carpenters (38) and floorlayers (33) |  | 71 males (?)  n/a |  | Video-recordings of main tasks at the workplace, 3-30 min each on one to six workers (total: 789 min for carpenters, 620 min for floorlayers) |  | Interviews immediately after the recordings to quantify (by %) the amount of time spent in kneeling, squatting and knee-supporting positions during the observed period) |  | n/a  Total percentage of video-recorded knee-straining work: 27%) |
|  |  |  |  |  |  |  |  |  |  |  |
| Klußmann et al. (2010) |  | Construction industry and pipework (e.g. painters, pavers, pipe layers, drivers) |  | 25 males  Median 42 (18-54 ) |  | Direct workplace observation, computer-assisted (handheld), full work shifts (with exceptions) |  | Questionnaire at the end of the observed shift (incl. different knee-straining postures, duration and frequency each) |  | 1.56 |
|  |  |  |  |  |  |  |  |  |  |  |
| Pope et al. (1998) |  | 6 settings (Supermarket, department store, packaging factory, post office, hospital, airport) |  | 123 (76 females and 47 males)  Mean 36 (SD n/a) |  | Direct workplace observation of major body postures, time sampling approach at 30-second intervals over one hour |  | Self-administered questionnaire (8 items on manual material handling, 4 items on work postures, 2 items on upper limb movements); kneeling: only qualitative assessed |  | n/a |
|  |  |  |  |  |  |  |  |  |  |  |
| Viikari-Juntura et al. (1996) |  | Forest industry (e.g. carpenters, repairmen, drivers, foremen) |  | 36 males  Mean 44.2 (SD 7.9) |  | Direct workplace observations, median duration 18 min (2-90 min), over several days |  | 1. Self-reports about an average work shift with 150 items, incl. 10 physical items (e.g. kneeling/squatting in h/day, 4 point ordinal scale)  2. Log books of 28 of 36 workers (not from the observed shift) |  | 0.00 |
